# Laboratory frailty index improves prediction of in-hospital falls among older adults

**DOI:** 10.1007/s40520-025-03090-9

**Published:** 2025-06-05

**Authors:** Hirotaka Nakashima, Takahiro Imaizumi, Hitoshi Komiya, Akemi Morohashi, Kazuhisa Watanabe, Chisato Fujisawa, Yosuke Yamada, Yoshimasa Nagao, Hiroyuki Umegaki

**Affiliations:** 1 Department of Geriatrics, Nagoya University Hospital, 65 Tsurumai-cho, Showa-ku, Nagoya, Aichi 464-8550 Japan; 2 Department of Advanced Medicine, Nagoya University Hospital, 65 Tsurumai-cho, Showa-ku, Nagoya, Aichi 464-8550 Japan; 3 Department of Patient Safety, Nagoya University Hospital, 65 Tsurumai- cho, Showa-ku, Nagoya, Aichi 464-8550 Japan; 4 Institute of Innovation for Future Society, Nagoya University, Furo-cho, Chikusa-ku, Nagoya, Aichi 464-8601 Japan

**Keywords:** Fall prediction, Fall screening, FI-lab, Frailty index-laboratory, STRATIFY

## Abstract

**Aims:**

To explore the association between Frailty Index based on laboratory tests (FI-lab) and in-hospital fall risk in older adults, and to explore whether incorporating FI-lab improves the predictive accuracy of a traditional fall risk prediction tool.

**Methods:**

We conducted a retrospective cohort study using electronic medical records from patients aged ≥ 60 years who were admitted to Nagoya University Hospital in 2020. We assessed fall risk using the St. Thomas’s Risk Assessment Tool in Falling Elderly Inpatients (STRATIFY). We calculated FI-lab based on 35 common laboratory parameters tested on admission. Each fall was reported prospectively by nurses through computer-based incident report forms. The relationship between FI-lab and in-hospital falls was analyzed using multivariate binomial logistic regression. Predictive performance was compared using the area under the receiver operating characteristic curve (AUROC) and net reclassification improvement (NRI). Missing data were not imputed and internal validation used 1000-bootstrap optimism-correction.

**Results:**

Data for 5984 patients were included (mean age 73 years, 63.5% male). The mean FI-lab score was 0.31 ± 0.16. Falls occurred in 175 patients (2.9%) during a median hospital stay of 9 days. FI-lab was associated with falls independently of STRATIFY. Adding FI-lab to STRATIFY significantly improved its predictive accuracy, increasing AUROC from 0.674 to 0.715 (*p* = 0.018), with NRI of 0.413 (*p* < 0.001). Calibration slope after internal validation was 0.97.

**Conclusions:**

FI-lab on admission was independently associated with in-hospital fall risk and improved the predictive ability of STRATIFY. FI-lab could be a valuable component in more accurate fall prediction.

**Supplementary Information:**

The online version contains supplementary material available at 10.1007/s40520-025-03090-9.

## Introduction

Falls are one of the most common accidents occurring in hospitals [1]. Falls are associated with trauma ranging from minor scratches to death and can impose a financial burden as a result of the additional medical care required and prolonged hospital stays [2, 3]. The risk of falls and potentially fatal fall-related injuries increases with age [4]. Furthermore, the problem of falls is escalating worldwide with population aging [5].

Various fall prediction tools have been developed [6, 7], but their predictive ability appears to be inadequate [6, 8]. Therefore, the search continues for more accurate prediction tools that are less invasive, time-consuming, and costly to use [9, 10].

Several abnormalities in laboratory tests have been reported to be associated with falls, including hyponatremia, anemia, and hypoalbuminemia [9, 11–13]. However, the existing fall prediction tools do not include laboratory test results [7]. Adding these results to the existing tools may improve their ability. A previous study found that addition of comorbidities, polypharmacy, number of drugs that increase the risk of falls, and laboratory test results improved the ability of the Morse Fall Scale to predict falls [9]. However, it is unclear whether adding laboratory tests alone to existing tools would improve their predictive ability.

The Frailty Index based on laboratory tests (FI-lab), proposed in 2014 as one of the indicators of frailty, essentially counts the number of laboratory test results that deviate from the normal range [14]. FI-lab is simple with high potential for automation [15] and is associated with mortality in various settings, including inpatient [16–18], home-based [19], nursing home [20], and community-based care [16, 21]. A previous study reported that FI-lab was associated with falls in community-dwelling older adults [21]. However, there are no reports on FI-lab and falls in the hospital setting. Hospitalized older adults tend to have more abnormal test results compared to community-dwelling older adults. Moreover, the interpretation of FI-lab may differ between these two groups. Specifically, FI-lab in hospitalized older adults may reflect acute disease states more strongly than in community-dwelling older adults. Therefore, it is essential to investigate the relationship between FI-lab and falls in hospitalized patients. Furthermore, no research has explored the impact of adding FI-lab to existing fall prediction tools. Such research is an important step toward clinical application.

The aforementioned previous study reported the effect of adding laboratory test results and other factors to the Morse Fall Scale in hospitalized older adults [9]. However, in our study, we could not calculate the Morse Fall Scale score from our hospital database. Instead, we were able to calculate the St. Thomas’s Risk Assessment Tool in Falling Elderly Inpatients (STRATIFY) score [22], a widely used and well-studied fall prediction tool in hospital settings [6, 7]. STRATIFY consists of five items: history of falls, mental status, vision, toileting, and mobility. Since STRATIFY does not incorporate laboratory test results, we hypothesized that combining STRATIFY with the FI-lab score could enhance its predictive ability.

The aims of this study were to determine whether FI-lab is associated with falls in hospitalized older adults and whether adding the FI-lab score can improve the predictive ability of STRATIFY, a traditional fall prediction tool.

## Methods

### Study design

This retrospective cohort study analyzed data extracted from the Nagoya University Hospital electronic medical records system. The study protocol was approved by the Ethics Committee of Nagoya University Hospital (approval number 2020 − 0357) and conducted in accordance with the principles of the Declaration of Helsinki and its amendments. The need for informed consent was waived in view of the analysis being based on patient data extracted from medical records. However, patients could withdraw from the study via the opt-out method by accessing the Nagoya University Hospital website in accordance with the national ethical guidelines for medical and health research involving human subjects [23]. The recommendations in the Strengthening the Reporting of Observational Studies in Epidemiology (STROBE) statement [24] and the Transparent Reporting of a multivariable prediction model for Individual Prognosis Or Diagnosis (TRIPOD) statement [25] were followed.

### Study population

Data for patients who met the following criteria were extracted from the medical records: admission between January 1, 2020 and December 31, 2020; age 60 years or older on admission; and hospitalization using the Diagnosis Procedure Combination (DPC)/Per-Diem Payment System (PDPS) [26]. The DPC/PDPS is a Japanese reimbursement system used in acute care hospitals in Japan, and excludes most psychiatric hospitalizations. For patients who were readmitted within 30 days of discharge, their readmission was excluded. Also, those who did not have sufficient laboratory data available to calculate an FI-lab score were excluded.

### Data collection

Demographic and anthropometric data were obtained from the medical records. Ability to perform activities of daily living on admission was evaluated by nurses using the Barthel Index [27], which is routinely recorded in the DPC/PDPS. The comorbidity burden was evaluated using the Charlson Comorbidity Index (CCI) [28]. The CCI score was calculated using the International Classification of Diseases, Tenth Revision (ICD-10) codes [29] extracted from the DPC/PDPS. Symptoms and signs on admission were recorded by nurses as part of routine clinical practice. Data on the use of medications on admission were also extracted, and the polypharmacy score [30] was calculated based on whether six medication categories were used (antihypertensive drugs, antidiabetics, antithrombotic drugs, sleep drugs, antipsychotics, and non-steroidal anti-inflammatory drugs). The polypharmacy score ranges from 0 to 6.

### Assessment of fall risk

Fall risk was assessed and recorded routinely on admission by nurses using the assessment tool originally developed by our hospital. This tool consists of 40 items and is used at our hospital to identify patients at risk of falling and intervene on this risk in routine clinical practice [30] but has not been adequately validated in the literature. In the present study, we calculated the STRATIFY [22] score using the extracted data. STRATIFY consists of five items, namely, history of falls, mental status, vision, toileting, and mobility. Each item has a score of 1, and the total STRATIFY score ranges from 0 to 5. We modified two items in STRATIFY to accommodate our hospital database and actual practice (Supplementary Table 1). We replaced the original STRATIFY “history of falls” item with our hospital’s fall history data, which is obtained through history-taking by nurses on admission, specifically regarding falls that occurred within one year. The “mental status” item in the original STRATIFY assesses agitation only. However, the frequency of agitation on admission was very low (0.6%) in our study population. Therefore, we used the modified criterion suggested by Papaioannou et al. [31], which includes confusion, disorientation, and agitation.

### Frailty indices

FI-lab contains 35 commonly measured laboratory parameters (Supplementary Table 2). The laboratory test data obtained on the day of admission or the following day were used. If the laboratory data were recorded more than once, the results of the first test were used. An FI-lab score was calculated for each patient by dividing the number of abnormal test results by the number of laboratory tests performed [14, 17]. The FI-lab score ranges from 0 to 1. We calculated the FI-lab score for patients who had at least 70% of laboratory results available. The measured ratio [32] was calculated by dividing the number of measured laboratory tests by 35 (the total number of laboratory tests).

We also generated a 40-item standard non-laboratory Frailty Index (FI-clinical) (Supplementary Table 3) based on the method proposed by Theou et al. [33] using the extracted data. Domains of FI-clinical include symptoms and signs, body mass index (BMI), comorbidities, functional ability, cognitive status, and sensory function. FI-clinical was calculated as the sum of the deficit values divided by the total number of items measured. FI-clinical ranges from 0 to 1, with a higher score indicating worse frailty. At least 80% of the items listed on FI-clinical were available in all participants, hence it was calculated among all of them.

### Outcome

The primary outcome was falls during hospitalization. A fall was defined as an unexpected event in which the patient came to rest on the ground, floor, or a lower level without known loss of consciousness. At our hospital, each fall is reported by a nurse using a standardized computer-based incident report form and confirmed by responsible staff (physicians, nurses, and a pharmacist) in the Department of Patient Safety.

The secondary outcomes were injurious falls during hospitalization, in-hospital mortality, length of hospital stay, and the rate of discharge to home. Injurious falls were defined as falls that resulted in abrasions, contusions, lacerations, bruises, sprains, pain, head injuries, other unspecified injuries, or any other serious injury.

Blinding and Timing of Predictor and Outcome Assessment.

All laboratory tests were conducted on the day of admission or the following day. Although a small proportion of falls may have occurred before blood collection, this is unlikely to have materially influenced the primary analysis. The STRATIFY, FI-lab, and FI-clinical scores were calculated later solely for research purposes. Falls were recorded prospectively during hospitalization by ward nurses, who were unaware of these predictor scores; therefore, outcome assessment was blinded to the predictors.

### Statistical analysis

The patient data were summarized using descriptive statistics. The baseline characteristics of excluded patients were compared with those of included patients using Fisher’s exact test, Student’s t-test, or Mann-Whitney U test, as appropriate. The included population was divided into three groups based on previously reported FI-lab cut-off points (< 0.25, 0.25–0.4, and > 0.4) [18]. Differences among the three groups were evaluated using Cochran-Armitage or Jonckheere-Terpstra tests for trends. Correlations between the FI-lab score and various clinical parameters, including age, BMI, the Barthel Index, CCI, STRATIFY score, and FI-clinical, were explored using Spearman’s rank correlation tests. Additionally, correlations between STRATIFY and FI-clinical were also explored.

We calculated odds ratios (ORs) and 95% confidence intervals (95% CIs) using a logistic regression model to assess the associations of in-hospital falls as the dependent variable with age, sex, STRATIFY, FI-clinical, and FI-lab scores as independent variables. In addition to analysis as a continuous variable, we also analyzed the FI-lab score as a binary variable with a cut-off point of 0.4 (≤ 0.4 or > 0.4). This cut-off point was set based on a previous report [18]. We calculated the ORs when the binary FI-lab variable was added to the five STRATIFY items, giving a total of six items in view of enhancing the clinical feasibility.

The ability of the STRATIFY, FI-lab, and FI-clinical used individually and in combination to predict falls was calculated by receiver operating characteristic (ROC) curve analysis. An area under the ROC curve (AUROC) of 0.5 indicates no discrimination and 1.0 indicates perfect discrimination. The AUROCs were compared using the DeLong method [34]. The risk reclassification ability of FI-lab and FI-clinical score was evaluated by the net reclassification improvement (NRI) and integrated discrimination improvement (IDI) indices. Internal validation of models was carried out using bootstrap methods (1000 resampling) in the rms packages version 8.0–0 in R version 4.5.0.

We conducted several subgroup analyses by dividing the patients by age (stratified at 75 years) and sex to determine whether these factors affect the association between the FI-lab score and the risk of falls. We also conducted four sensitivity analyses. The first analysis included the polypharmacy score as a covariate. The second analysis included the CCI score as a covariate. The third analysis employed ROC curve analysis using the Delong method to determine a modified FI-lab cut-off point for in-hospital falls. The fourth analysis utilized our hospital’s fall risk assessment tool [30] instead of STRATIFY. Our assessment tool consists of seven categories, including history of falls, activities of daily living, cognitive function, sensory function, and medication (Supplementary Table 4). Each category contains 2 to 7 items, and the total number of items is 40. A score of 1 is assigned to a category if any item in the category is checked “yes.” The sum of the scores for each category is defined as the fall risk score and ranges from 0 to 7.

Statistical analyses other than internal validity were performed using EZR version 1.68 (Saitama Medical Center, Jichi Medical University, Saitama, Japan). EZR is a graphical user interface for R version 4.3.1 (The R Foundation for Statistical Computing, Vienna, Austria) [35]. A sample size calculation was not conducted a priori for this study, and all available data were used. A total of 175 fall events were available for analysis and the final multivariable model included four predictors (age, sex, STRATIFY score, FI-lab), giving an events-per-variable of 175 / 4 = 43.8, which exceeds widely recommended minimum thresholds. The analyses were performed without imputation of missing values. A p-value of < 0.05 was considered statistically significant.

## Results

A total of 7661 patients aged ≥ 60 years were admitted to our hospital via the DPC/PDPS during the study period. After exclusion of 1677 patients because of readmission within 30 days (*n* = 1468), insufficient laboratory data (*n* = 176), or both (*n* = 33), data for 5984 patients were analyzed. Patient ages ranged from 60 to 99 years (mean 73.0, SD 7.3 years) and 63.5% were male (Table 1). The median STRATIFY score was 0 (IQR 0, 1), the mean FI-lab score was 0.31 ± 016, and the mean FI-clinical score was 0.11 ± 0.09. The frequency distributions for the STRATIFY, FI-lab, and FI-clinical scores are shown in Supplementary Fig. 1. The most common primary diagnoses prompting admission are shown in Supplementary Table 5. The 1,677 excluded patients were younger, had better functional ability (Barthel Index), a higher disease burden (CCI), and a lower proportion of emergency admissions compared to the included patients (Supplementary Table 6). These results suggest that many of excluded hospitalizations were repeated planned hospitalizations for conditions such as cancer.

**Table 1:** Baseline characteristics of the study population and outcomes

Characteristics and outcomes	Value	
Participants, n (%)	5984	(100.0)
Age, years	73.0	± 7.3
Male sex	3798	(63.5)
Emergency hospitalization	1551	(25.9)
Body mass index, kg/m^2^	22.3	± 3.8
Barthel Index^a^, median (IQR)	100	(100–100)
Barthel Index^a^, mean ± SD^b^	94.1	± 15.5
CCI^c^	2	(1–2)
Polypharmacy score^d^	1	(0–2)
STRATIFY score^e^, median (IQR)	0	(0–1)
STRATIFY score^e^, mean ± SD^b^	0.42	± 0.72
History of falls	807	(13.5)
Mental state abnormality	353	(5.9)
Vision impairment	442	(7.4)
Frequent urination	419	(7.0)
Transferring and/or mobility impairment	504	(8.4)
FI-lab^f^	0.31	± 0.16
Measured ratio	0.87	± 0.08
FI-clinical^f^	0.11	± 0.09
Outcomes		
In-hospital falls	175	(2.9)
In-hospital injurious falls	32	(0.5)
In-hospital mortality	95	(1.6)
Length of hospital stay	9	(5–16)
Discharge home	5382	(89.9)

*Notes*: Data are presented as the mean ± standard deviation, median (interquartile range), or number (percentage). CCI = Charlson Comorbidity Index; FI-clinical = standard non-laboratory Frailty Index; FI-lab = Frailty Index based on laboratory tests; IQR = interquartile range; SD = standard deviation; STRATIFY = St. Thomas’s Risk Assessment Tool in Falling Elderly Inpatients

^a^Barthel Index score ranges from 0 to 100, with a higher score indicating higher function

^b^The mean and SD are shown for reference

^c^The CCI score ranges from 0 to 37, with a higher score indicating more comorbidities

^d^The polypharmacy score ranges from 0 to 6, with a higher score indicating more medications in use

^e^The STRATIFY score ranges from 0 to 5, with a higher score indicating a higher fall risk

^f^The FI-lab and FI-clinical scores range from 0 to 1, with a higher score indicating worse frailty

Comparisons among the three FI-lab levels are shown in Supplementary Table 7. The groups with higher FI-lab scores were significantly older, had lower BMI, lower Barthel Index, higher CCI, higher STRATIFY score, and higher FI-clinical score. However, correlations between FI-lab and these parameters were weak (age, *r* = 0.17; BMI, *r*=–0.13; Barthel Index, *r*=–0.29; CCI, *r* = 0.20; STRATIFY, *r* = 0.17; FI-clinical, *r* = 0.37; all *p* < 0.001). On the other hand, there was a moderate correlation between STRATIFY and FI-clinical (*r* = 0.66, *p* < 0.001).

**Fig. 1:**
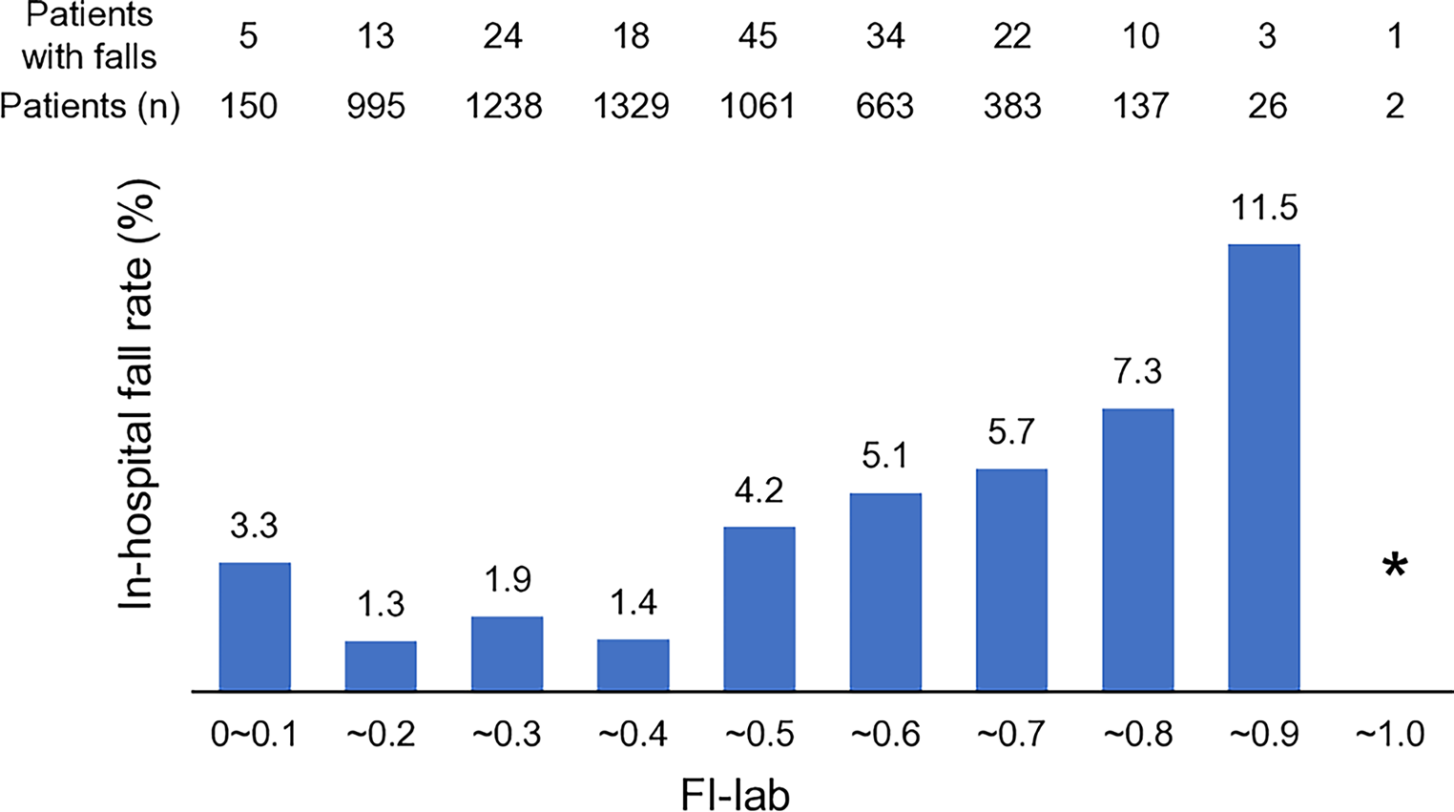
Rate of falls by FI-lab score (*n* = 5984). Patients with a higher Frailty Index-laboratory (FI-lab) score had a higher fall rate. FI-lab score ranges from 0 to 1, with a higher score indicating worse frailty. The bar for FI-lab between 0.9 and 1.0 (indicated by *) is not drawn because the number of patients was too small (*n* = 2). (Alt text: Bar chart showing the relationship between the Frailty Index laboratory and fall rates during hospitalization. As the Frailty Index laboratory score increases, the fall rate also increases.)

A total of 175 patients (2.9%) sustained falls during hospitalization (Table 1). Patients with a higher FI-lab score had a higher fall rate (Fig. 1). The results of the multiple regression analyses for in-hospital falls are shown in Table 2. FI-lab was associated with in-hospital falls independently of STRATIFY (model 2, FI-lab per 0.1 unit, adjusted OR 1.28, 95% CI 1.16–1.40). Similarly, FI-clinical was associated with in-hospital falls independently of STRATIFY (model 4, FI-clinical per 0.1 unit, adjusted OR 1.40, 95% CI 1.19–1.65). When STRATIFY, FI-lab and FI-clinical were simultaneously included as covariates, the two frailty indices were independently associated with in-hospital falls (model 6). Full model coefficients, including intercept, are shown in Supplementary Table 8.

**Table 2:** Univariate and multivariate logistic regression analyses for in-hospital falls (*n* = 5984)

	Univariate OR	Multivariate^d^					
		Model 1	Model 2	Model 3	Model 4	Model 5	Model 6
		OR	OR	OR	OR	OR	OR
Variable	(95% CI)	(95% CI)	(95% CI)	(95% CI)	(95% CI)	(95% CI)	(95% CI)
STRATIFY^a^	1.89	1.82	1.65		1.35		1.37
	(1.64–2.19)	(1.57–2.12)	(1.41–1.93)		(1.08–1.67)		(1.11–1.70)
FI-lab^b^	1.36		1.28			1.23	1.23
	(1.25–1.49)		(1.16–1.40)			(1.12–1.35)	(1.12–1.36)
STRATIFY	1.86			1.81			
including FI-lab^c^	(1.65–2.09)			(1.60–2.05)			
FI-clinical^b^	1.68				1.40	1.48	1.25
	(1.51–1.87)				(1.19–1.65)	(1.31–1.68)	(1.05–1.49)

*Notes*: CI = confidence interval; FI-clinical = standard non-laboratory Frailty Index; FI-lab = Frailty Index-laboratory; OR = odds ratio; STRATIFY = St. Thomas’s Risk Assessment Tool in Falling Elderly Inpatients

^a^The STRATIFY score ranges from 0 to 5, with a higher score indicating a higher falls risk. Odds ratios per point are shown

^b^The FI-lab and FI-clinical scores range from 0 to 1, with a higher score indicating more severe frailty. Odds ratios per 0.1 unit are shown

^c^Total of 6 items: 5 STRATIFY items and 1 item based on whether the FI-lab score was higher than 0.4. Odds ratios per point are shown

^d^Each model consists of age, sex, and items with values described

**Fig. 2:**
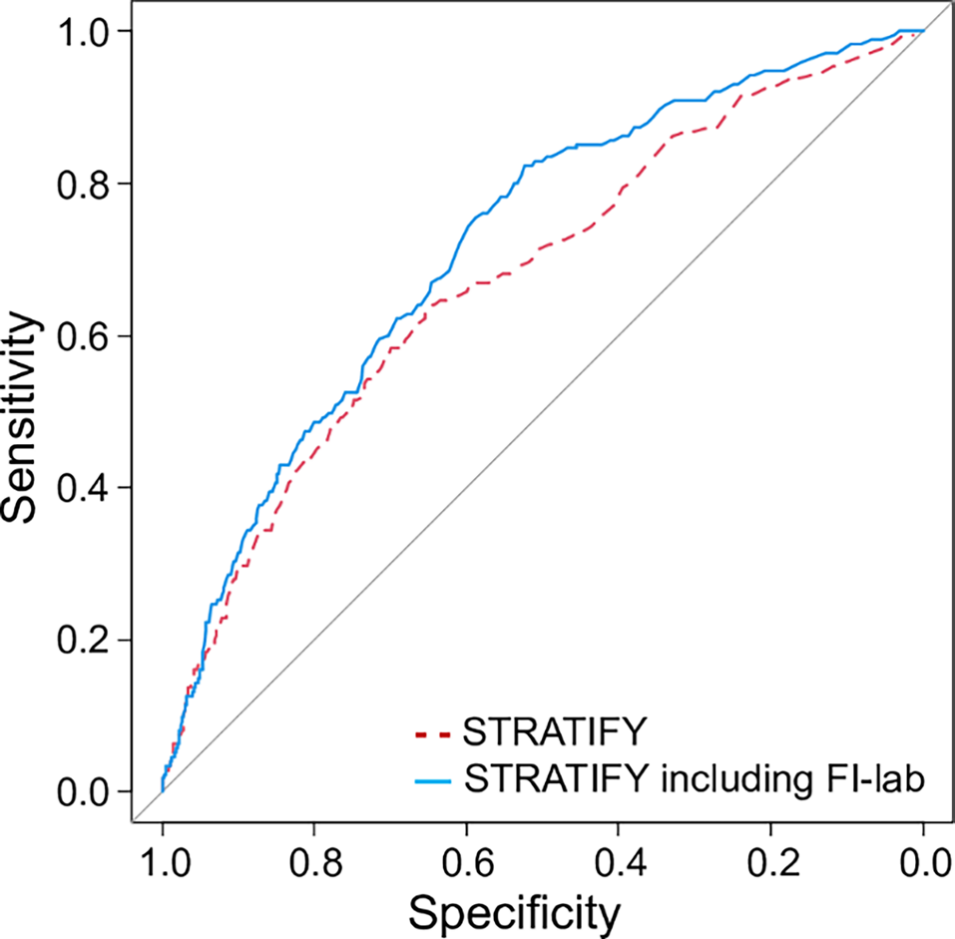
Receiver operating characteristic curves for the STRATIFY score alone and the STRATIFY score including FI-lab for in-hospital falls (*n* = 5984). The STRATIFY (St. Thomas’s Risk Assessment Tool in Falling Elderly Inpatients) score is a traditional fall prediction tool composed of five items and ranges from 0 to 5. “STRATIFY including FI-lab” includes the five STRATIFY items and one binary item based on whether Frailty Index-laboratory (FI-lab) was higher than 0.4. Area under the receiver-operating characteristic curve of STRATIFY including FI-lab (0.718) was significantly higher than that of STRATIFY alone (0.674) (*p* < 0.001). (Alt text: The figure shows two receiver operating characteristic curves representing the performance of predicting falls in hospitalized patients. One curve demonstrates the performance of the STRATIFY, a traditional fall prediction tool, while the other shows the improved performance when the Frailty Index laboratory is added)

Table 3 shows the predictive performance of STRATIFY, FI-lab, and FI-clinical when used individually and in combination. Adding FI-lab to STRATIFY (model 2) significantly increased the AUROC from 0.674 to 0.715 (*p* = 0.018), with a significant NRI of 0.413 (95% CI 0.265–0.560, *p* < 0.001), indicating that the new model improved the risk reclassification for 41.3% of patients. The IDI was 0.005 (95% CI 0.002–0.008, *p* < 0.001), indicating a small but significant improvement in the overall discriminative ability of the model. The results were similar when the FI-lab score was converted to a binary variable and added to STRATIFY (model 3; AUROC = 0.718, comparison with STRATIFY alone, *p* < 0.001) (Fig. 2). Adding FI-clinical increased the predictive performance of STRATIFY (model 4; AUROC 0.707, 95% CI 0.667–0.747, difference *p* < 0.001; NRI 0.173, 95% CI 0.023–0.323, *p* = 0.024) although IDI was not statistically significant (IDI 0.002, 95% CI -0.0002–0.005, *p* = 0.08). Concomitant use of FI-lab and FI-clinical resulted in a higher AUROC (model 5; AUROC = 0.726) compared to STRATIFY alone (difference *p* = 0.011). NRI and IDI for model 5 were not calculated because this model did not include STRATIFY. The combination of all three scores (STRATIFY, FI-lab, and FI-clinical) resulted in the highest AUROC, with a significant NRI and IDI (model 6; AUROC = 0.733; NRI = 0.400; IDI = 0.006).

**Table 3:** Predictive performance of STRATIFY, FI-lab, and FI-clinical for in-hospital falls (*n* = 5984)

Model	Variable^a^	AUC	95% CI	*p*	NRI	95% CI	*p*	IDI	95% CI	*p*
Model 1	STRATIFY	0.674	(0.632–0.716)	ref	ref		ref	ref		ref
Model 2	STRATIFY	0.715	(0.677–0.753)	0.018	0.413	(0.265–0.560)	< 0.001	0.005	(0.002–0.008)	< 0.001
	+ FI-lab									
Model 3	STRATIFY	0.718	(0.681–0.755)	< 0.001	0.519	(0.369–0.668)	< 0.001	0.005	(0.003–0.008)	< 0.001
	Including FI-lab^b^									
Model 4	STRATIFY	0.707	(0.667–0.747)	0.003	0.173	(0.023–0.323)	0.024	0.002	(-0.0002–0.005)	0.08
	+ FI-clinical									
Model 5	FI-lab	0.726	(0.690–0.763)	0.011	n/a			n/a		
	+ FI-clinical									
Model 6	STRATIFY	0.733	(0.697–0.769)	< 0.001	0.400	(0.248–0.551)	< 0.001	0.006	(0.002–0.009)	0.002
	+ FI-lab									
	+ FI-clinical									

*Notes*: AUC = area under the receiver-operating characteristic curve; CI = confidence interval; FI-clinical = standard non-laboratory Frailty Index; FI-lab = Frailty Index based on laboratory tests; IDI = integrated discrimination improvement; NRI = net reclassification improvement; STRATIFY = St. Thomas’s Risk Assessment Tool in Falling Elderly Inpatients

^a^All models include age and sex as covariates

^b^Total of 6 items: 5 STRATIFY items and 1 item based on whether the FI-lab score was higher than 0.4

Internal validation (Supplementary Table 9) showed that the optimism-corrected c-statistics were similar to the original AUCs, and the models’ calibration slopes were close to 1. Additionally, the mean absolute error ranged from 0.004 to 0.008, and the 0.9 quantile of absolute error ranged from 0.009 to 0.016.

The results of the subgroup and sensitivity analyses are shown in Supplementary Tables 10 and 11. Adding FI-lab to STRATIFY significantly improved the AUROC regardless of age (stratified at 75 years) and sex. Adding polypharmacy or the CCI score did not impact the results. Analysis of the AUROCs revealed that an FI-lab cut-off point of 0.345 had the best ability to predict in-hospital falls. However, changing the FI-lab cut-off point from 0.40 to 0.345 did not significantly improve the AUROC whether the binary FI-lab variable was used alone or included in STRATIFY. Finally, the fall risk assessment tool used at our hospital predicted in-hospital falls with accuracy similar to that of STRATIFY, and addition of the binary FI-lab variable (cut-off 0.40) to the fall risk assessment tool significantly improved the predictive ability.

The results for the secondary outcomes are shown in Table 1 and Supplementary Table 7. Patients with a higher FI-lab score more frequently sustained injurious falls, but this finding was not statistically significant.

## Discussion

In this retrospective cohort study, the FI-lab score on admission was associated with the risk of in-hospital falls independently of the STRATIFY score. Furthermore, adding the FI-lab score to the STRATIFY score improved predictive ability. Similarly, the FI-clinical score was independently associated with in-hospital falls, and incorporating it into the STRATIFY score improved predictive ability. The combined use of both the FI-lab and FI-clinical scores, even without the STRATIFY score, demonstrated superior predictive ability compared to STRATIFY alone.

When interpreting the results of this study, it is important to consider what the FI-lab is assessing in patients. We found that the correlation between the FI-lab score and the Barthel Index was weak, which is consistent with previous findings in older adults receiving inpatient [17] or home-based medical care [19]. This weak association is not surprising given that FI-lab does not assess the patient’s functional status. In view of these results and the nature of FI-lab, it appears that the FI-lab evaluates the accumulated acute or chronic organ dysfunction reflected in laboratory tests. Therefore, the FI-lab score may serve as an indicator of multimorbidity, which is a known risk factor for falls in older adults [36]. STRATIFY, which does not account for comorbidity, was used as the traditional fall prediction tool in the present study. FI-lab may have improved the predictive performance by compensating for the inability of STRATIFY to evaluate multimorbidity. Additionally, we also assessed comorbidities using CCI, and the correlation between the CCI and the FI-lab score was weak in this study, which is consistent with previous findings [17, 19]. This weak correlation may reflect the fact that the CCI includes a number of conditions that cannot be detected by blood tests (e.g., stroke).

We found only one previous study that evaluated the association between the FI-lab score and falls [21]. In that study, falls were assessed as a secondary outcome in 2933 community-dwelling adults with a mean age of 60.2 years. The FI-lab score was calculated based on 23 common blood tests and vital signs and found to have an average value of 0.28. The FI-lab score was associated with falls (FI-lab per 0.01 unit, adjusted for age, OR 1.01, 95% CI 1.00–1.02). Despite differences in the study populations and the items included in FI-lab tool, the results of that study are consistent with our present findings. Furthermore, in the present study, the FI-lab score was associated with falls independent of a traditional fall prediction tool.

Interestingly, the fall rate in the group with the lowest FI-lab (FI-lab 0.0–0.1, fall rate 3.3%) was higher than that in the group with slightly higher FI-lab (FI-lab 0.1–0.2, fall rate 1.3%). This might be due to the relatively small sample size in the lowest FI-lab group, where the impact of a single fall could be greater. Alternatively, it could suggest that patients with lower FI-lab scores, which indicate minimal organ dysfunction, had greater physical activity and mobility, leading to a higher risk of falls.

FI-clinical was associated with in-hospital falls, consistent with previous reports [37]. Adding FI-clinical to STRATIFY significantly improved AUROC and NRI, but not IDI (model 4). In contrast, adding FI-lab (model 2) significantly improved all of AUROC, NRI, and IDI. Furthermore, the lower limit of the CI for NRI with FI-lab (model 2, 0.265) was higher than with FI-clinical (model 4, 0.023), suggesting that the improvement in predictive ability was greater with FI-lab than with FI-clinical. This may be due to the overlap of the items that constitute FI-clinical and STRATIFY, which were moderately correlated (*r* = 0.66).

Notably, the combined use of FI-clinical and FI-lab without STRATIFY (model 5) had higher predictive ability than STRATIFY alone. Some places have a system to automatically calculate FI-clinical using information in electronic medical records [38]. In such situations, the combined use of FI-clinical and FI-lab without specific fall prediction tool may be applicable.

The implications of this study are that, in hospitals that use STRATIFY as a fall prediction tool, adding FI-lab may improve its predictive ability, and that the predictive ability of other fall prediction tools might also be improved by addition of FI-lab. The FI-lab score could also improve predictive tools for conditions other than falls. In our study, the predictive ability was unaffected by whether the FI-lab score was treated as a continuous variable or as a binary variable. FI-lab may be easier to implement in clinical practice if used as a binary variable.

Although adding the FI-lab score to the STRATIFY score significantly improved the ability to predict falls, the resulting AUROC of 0.715 (model 2) was still relatively low. Both the present results and existing fall prediction tools generally show relatively low accuracy [6, 8]. Consequently, recent guidelines recommend against using a specific fall prediction scoring system [8]. However, adding new components to fall prediction tools could improve their predictive ability enough to be of practical use. In the present study, we incorporated information on multimorbidity using the FI-lab score. The CCI may reflect comorbidities that cannot be assessed by the FI-lab, and adding the CCI was expected to further improve predictive ability. However, this was not supported by the sensitivity analysis (Supplementary Table 11). Since the CCI in this study was calculated from data extracted from DPC/PDPS, there may have been insufficient identification of comorbidities. A more accurate comorbidity indicator might improve predictive ability. Other potential components include more refined indicators of polypharmacy [39] and direct observations of mobility [30]. Further research is needed to develop more accurate prediction models. Even if accurate fall prediction tools are developed, patient education, as well as detailed assessment and intervention for high-risk patients, will remain important. Nevertheless, this study is the first to evaluate the usefulness of including the FI-lab score in a clinical prediction tool.

The strengths of this study include its relatively large sample size. Additionally, the results obtained using STRATIFY were similar to those obtained using our hospital’s fall prediction tool. Another key strength is the confirmation of internal validity. This study also has some limitations. First, it had a retrospective cohort design, although the falls data were collected prospectively. Second, this is a single-site study. Caution is needed when generalizing its findings to other institutions and regions. Third, we excluded patients with insufficient laboratory data. Although the number of these patients was not large, imputation of missing data could have further increased the robustness of the results.

## Conclusion

The FI-lab score on admission was associated with the risk of in-hospital falls independently of the STRATIFY score, a widely used fall prediction tool. Addition of the FI-lab score to the STRATIFY score improved the predictive ability for in-hospital falls. Further studies in other settings are needed.

## Electronic supplementary material

Below is the link to the electronic supplementary material.

Supplementary Material 1

## Data Availability

No datasets were generated or analysed during the current study.
