# Supplementary material for: Laboratory frailty index improves prediction of in-hospital falls among older adults
Source: Aging Clin Exp Res. 2025 Jun 5;37(1):179. doi: 10.1007/s40520-025-03090-9 (PMC12141401; doi:10.1007/s40520-025-03090-9)
Supplement: Supplementary file 1 — Supplementary Material 1 [file 40520_2025_3090_MOESM1_ESM.pdf]

Supplementary Information

Article title

Laboratory frailty index improves prediction of in-hospital falls among older adults

Journal name

Aging Clinical and Experimental Research

Authors

Hirotaka Nakashima, Takahiro Imaizumi, Hitoshi Komiya, Akemi Morohashi, Kazuhisa Watanabe, Chisato Fujisawa, Yosuke Yamada, Yoshimasa Nagao, Hiroyuki Umegaki

***Corresponding author**

Hirotaka Nakashima

Department of Geriatrics, Nagoya University Hospital, Nagoya, Japan

Email: nakashima.hirotaka.d5@f.mail.nagoya-u.ac.jp

Supplementary Table 1. Original version of STRATIFY and the modified versions used in the present study.

Supplementary Table 2. Laboratory data used to construct the Frailty Index-laboratory (FI-lab).

Supplementary Table 3. Items included in the standard non-laboratory Frailty Index (FI-clinical).

Supplementary Table 4. Fall risk assessment tool used at the authors' hospital.

Supplementary Table 5. Common primary reasons for admission.

Supplementary Table 6. Comparison of baseline characteristics between included and excluded patients

Supplementary Table 7. Baseline characteristics of the study population and outcomes according to FI-lab score (n=5984).

Supplementary Table 8. Regression coefficients and standard errors for predictors in six prognostic models

Supplementary Table 9. Internal validation results of the model using bootstrap resampling (1000 times)

Supplementary Table 10. Results of subgroup analyses.

Supplementary Table 11. Results of sensitivity analyses.

Supplementary Figure 1. Frequency distributions for the STRATIFY (A), standard non-laboratory Frailty Index (FI-clinical) (B), and Frailty Index-laboratory (FI-lab) (C) (n=5984).

Supplementary Material. TRIPOD Checklist

Supplementary Table 1

Original version of STRATIFY and the modified versions used in the present study.

	Item	Original STRATIFY*	Modified STRATIFY
1	History of falls	Did the patient present to hospital with a fall or has he or she fallen on the ward since admission? (Yes = 1, No = 0)	History of falls within 1 year.‡ (Yes = 1, No = 0)
2	Mental status	Agitated?† (Yes = 1, No = 0)	Is the patient confused, disoriented, or agitated?†,§ (Yes = 1, No = 0)
3	Vision	Visually impairment to the extent that everyday function is affected?† (Yes = 1, No = 0)	(Same as the original)
4	Toileting	In need of especially frequent toileting?† (Yes = 1, No = 0)	(Same as the original)
5	Transfer and mobility	Transfer and mobility score of 3 or 4?† (Yes = 1, No = 0)	(Same as the original)

Notes: STRATIFY = St. Thomas's Risk Assessment Tool in Falling Elderly Inpatients

*Based on Oliver et al. BMJ 1997; 315: 1049.

†Judged by nurses. No attempt was made to standardize the formation of these judgments.

‡Based on history-taking.

§Referring to Papaioannou et al. BMC Medicine 2004; 2: 1.

Supplementary Table 2

Laboratory data used to construct the Frailty Index-laboratory (FI-lab).

Variable*	Unit	Total population		Male		Female	
		0 (Normal)	1 (Abnormal)	0 (Normal)	1 (Abnormal)	0 (Normal)	1 (Abnormal)
WBC	$\times 10^3$/μL	3.3-8.6	<3.3 or >8.6				
Neutrophil	$\times 10^3$/μL	1.0-7.5	<1.0 or >7.5				
Lymphocyte	$\times 10^3$/μL	1.0-4.0	<1.0 or >4.0				
Hemoglobin	g/dL			13.7-16.8	<13.7 or >16.8	11.6-14.8	<11.6 or >14.8
Hematocrit	%			40.7-50.1	<40.7 or >50.1	35.1-44.4	<35.1 or >44.4
MCV	fL	83.6-98.2	83.6< or >98.2				
RDW	%	11-14	<11 or >14				
Platelet count	$\times 10^3$/μL	158-348	<158 or >348				
Total protein	g/dL	6.6-8.1	<6.6 or >8.1				
Albumin	g/dL	4.1-5.1	<4.1 or >5.1				
Glucose	mg/dL	73-109	<73 or >109				
Urea	mg/dL	8-20	<8 or >20				
Creatinine	mg/dL			0.65-1.07	<0.65 or >1.07	0.46-0.79	<0.46 or >0.79
Uric acid	mg/dL			3.7-7.8	<3.7 or >7.8	2.6-5.5	<2.6 or >5.5
Sodium	mmol/L	138-145	<138 or >145				
Potassium	mmol/L	3.6-4.8	<3.6 or >4.8				
Chloride	mmol/L	101-108	<101 or >108				
Corrected calcium	mg/dL	8.8-10.1	<8.8 or >10.1				
AST	U/L	13-30	<13 or >30				
ALT	U/L			10-42	<10 or >42	7-23	<7 or >23
LDH	U/L	124-222	<124 or >222				
Total bilirubin	mg/dL	0.4-1.5	<0.4 or >1.5				
ALP (JSCC)	U/L	106-322	<106 or >322				
γGTP	U/L			13-64	<13 or >64	9-32	<9 or >32
Amylase	U/L	44-132	<44 or >132				
CK	U/L			59-248	<59 or >248	41-153	<41 or >153
Total cholesterol	mg/dL	142-248	<142 or >248				
Triglyceride	mg/dL			40-234	<40 or >234	30-117	<30 or >117
HDL-C	mg/dL			38-90	<38 or >90	48-103	<48 or >103
LDL-C	mg/dL	65-163	<65 or >163				
HbA1c	%	4.9-6.0	<4.9 or >6.0				
CRP	mg/dL	≤0.14	>0.14				
PT	%	80-120	<80 or >120				
APTT	%	80-120	<80 or >120				
Fibrinogen	mg/dL	200-400	<200 or >400				

Notes: ALT = alanine transaminase; ALP = alkaline phosphatase; APTT = activated partial thromboplastin time; AST = aspartate transaminase; CK = creatine kinase; CRP = C-reactive protein; γGTP = gamma-glutamyl transpeptidase; HbA1c = hemoglobin A1c; HDL-C = high-density lipoprotein cholesterol; JSCC = Japan Society of Clinical Chemistry; LDH = lactate dehydrogenase; LDL-C = low-density lipoprotein-cholesterol; MCV = mean cell volume; PT = prothrombin time; RDW = red cell distribution width; WBC = white blood cells

*Cut-off values were obtained from our Department of Laboratory Medicine.

Supplementary Table 3

Items included in the standard non-laboratory Frailty Index (FI-clinical).

Variable		Deficit value				
		0 points	0.33 points	0.5 points	0.66 points	1 point
1	History of falls	absent				present
2	Muscle weakness	absent				present
3	Difficulty with balance	absent				present
5	Shuffling or festinating gate	absent				present
4	Contracture	absent				present
6	Frequent urination	absent				present
7	Nocturia	absent				present
8	BMI	21.5-25.0 kg/m^2				<21.5 or >25.0 kg/m^2
9	Coronary artery disease	absent				present
10	Congestive heart failure	absent				present
11	Peripheral artery disease	absent				present
12	Dementia	absent				present
13	COPD	absent				present
14	Peptic ulcer disease	absent				present
15	Chronic kidney disease	absent				present
16	Cerebral vascular accident	absent				present
17	Diabetes mellitus	absent				present
18	Hypertension	absent				present
19	Use of sleep medication(s)	no				yes
20	Feeding	independent		needs help		totally dependent
21	Position change in bed	independent		needs help		totally dependent
22	Maintaining sitting position	independent				totally dependent
23	Sitting adjustment	independent				totally dependent
24	Transfer	independent	minor help		major help	totally dependent
25	Walking	independent	walks with help		wheelchair bound	Immobile
26	Use of walking aids	no				yes
27	Stairs	independent		needs help		total dependent
28	Grooming	independent				needs help with personal care
29	Bathing	independent				total dependent
30	Dressing	independent		needs help		total dependent
31	Toilet	independent		needs help		totally dependent
32	Bowel control	continent		occasional accident		incontinent
33	Use of diaper for defecation	no				yes
34	Bladder control	continent		occasional accident		incontinent
35	Use of diaper for urination	no				yes
36	Disorientation	absent				present
37	Impaired comprehension	absent				present
38	Impaired memory	absent				present
39	Swallowing difficulty	absent				present
40	Hearing problems	no problems				hear only loud voices

Supplementary Table 4

Fall risk assessment tool used at the authors' hospital.

Major category	Items
1. History of falls	
	1. History of falls
	2. History of syncope
	3. History of convulsions
	4. History of epilepsy
2. Disorder of the sensory system	
	1. Visual impairment
	2. Hearing impairment
3. Disorder of the musculoskeletal system	
	1. Muscle weakness
	2. Paralysis, numbness
	3. Dizziness
	4. Lightheadedness
	5. Joint abnormalities/contractures/deformations
	6. Gait disturbance
4. Disorder of cognition function	
	1. Disturbance of consciousness
	2. Restlessness
	3. Disorientation
	4. Memory disturbance
	5. Impaired judgment
	6. Not able to press a buzzer
5. Dysfunction of urination/defecation	
	1. Needs helper
	2. Fecal incontinence
	3. Frequent urination
	4. Go to bathroom often at night
	5. Needs diapers for defecation
	6. Urinary incontinence
	7. Needs diapers for urination
6. Mobility dysfunction/dependency	
	1. Inability to stand or walk
	2. Cane
	3. Walker
	4. Wheelchair
	5. Need assistance
	6. Use of intravenous infusion, placement of drains, oxygenation
7. Medication	
	1. Hypnotics (occasionally)
	2. Hypnotics (every day)
	3. Psychotropic drugs
	4. Anticancer agents
	5. Opioids
	6. Analgesics
	7. Antihypertensive agents

Supplementary Table 5

Common primary reasons for admission.

Rank	Major diagnostic category	n	%*
1	Cancer and complications	2650	44.3
2	Arrythmia	263	4.4
3	Heart failure	212	3.5
4	Arterial diseases	189	3.2
5	Neurological diseases other than stroke	175	2.9
6	Benign gastrointestinal tumor	156	2.6
7	Renal failure	130	2.2
8	Biliary diseases	130	2.9
9	Cardiac valvular diseases	110	1.8
10	Diabetes mellitus and complications	99	1.7
11	Acute coronary syndrome	82	1.4
12	Pneumonia	65	1.1
13	Gastrointestinal ileus	60	1.0
14	Gastrointestinal bleeding	57	1.0
15	Autoimmune disorders	45	0.8

*Percentage of cases to total admissions (n=5984).

Supplementary Table 6

Comparison of baseline characteristics between included and excluded patients

Characteristics and outcomes	Included		Excluded		p[h]
Number of patients, n	5984		1677		
Age, years	73.0	± 7.3	71.7	± 6.7	<0.001[i]
Male sex	3798	(63.5)	990	(59.0)	0.001[j]
Emergency hospitalization	1551	(25.9)	252	(15.0)	<0.001[j]
Body mass index, kg/m^2	22.3	± 3.8	21.8	± 3.9	<0.001[i]
Barthel Index[a], median (IQR)	100	(100–100)	100	(100–100)	<0.001[k]
Barthel Index[a], mean ± SD[b]	94.1	± 15.5	96.5	± 12.2	<0.001[i]
CCI[c]	2	(1–2)	2	(2–2)	<0.001[k]
Polypharmacy score[d]	1	(0–2)	1	(0–2)	0.008[k]
STRATIFY score[e], median (IQR)	0	(0–1)	0	(0–1)	0.003[k]
STRATIFY score[e], mean ± SD[b]	0.42	± 0.72	0.35	± 0.64	<0.001[i]
History of falls	807	(13.5)	227	(13.5)	0.97[j]
Mental state abnormality	353	(5.9)	69	(4.1)	0.004[j]
Vision impairment	442	(7.4)	96	(5.7)	0.020[j]
Frequent urination	419	(7.0)	115	(6.9)	0.87[j]
Transferring and/or mobility impairment	504	(8.4)	85	(5.1)	<0.001[j]
FI-lab[f]	0.31	± 0.16	0.34	± 0.15[g]	<0.001[i]
Measured ratio	0.87	± 0.08	0.77	± 0.18	<0.001[i]
FI-clinical[f]	0.11	± 0.09	0.10	± 0.08	<0.001[i]

Notes: Data are presented as the mean ± standard deviation, median (interquartile range), or number (percentage). CCI = Charlson Comorbidity Index; FI-clinical = standard non-laboratory Frailty Index; FI-lab = Frailty Index based on laboratory tests; IQR = interquartile range; SD = standard deviation; STRATIFY = St. Thomas's Risk Assessment Tool in Falling Elderly Inpatients.

[a]Barthel Index score ranges from 0 to 100, with a higher score indicating higher function.

[b]The mean and SD are shown for reference.

[c]The CCI score ranges from 0 to 37, with a higher score indicating more comorbidities.

[d]The polypharmacy score ranges from 0 to 6, with a higher score indicating more medications in use.

[e]The STRATIFY score ranges from 0 to 5, with a higher score indicating a higher fall risk.

[f]The FI-lab and FI-clinical scores range from 0 to 1, with a higher score indicating worse frailty.

[g]n=1468

[h]Comparison between the two groups.

[i]Student's t-test.

[j]Fisher's exact test.

[k]Mann-Whitney U test.

Supplementary Table 7

Baseline characteristics of the study population and outcomes according to FI-lab score (n=5984).

	Total		Low (<0.25)		Moderate (0.25–0.4)		High (>0.4)		p[g]
Participants, n (%)	5984	(100.0)	2383	(39.8)	1859	(31.1)	1742	(29.1)	
Age, years	73.0	± 7.3	71.5	± 6.7	73.7	± 7.3	74.4	± 7.7	<.001[h]
Male sex	3798	(63.5)	1383	(58.0)	1253	(67.4)	1162	(66.7)	<.001[i]
Emergency hospitalization	1551	(25.9)	236	(9.9)	395	(21.2)	920	(52.8)	<.001[i]
Body mass index, kg/m^2	22.3	± 3.8	22.8	± 3.5	22.3	± 3.9	21.8	± 4.0	<.001[h]
Barthel Index[a], median (IQR)	100	(100–100)	100	(100–100)	100	(100–100)	100	(80–100)	<.001[h]
Barthel Index[a], mean ± SD[b]	94.1	± 15.5	98.0	± 8.2	95.4	± 13.3	87.4	± 21.7	
CCI[c]	2	(1–2)	2	(0–2)	2	(1–2)	2	(1–3)	<.001[h]
Polypharmacy score[d]	1	(0–2)	1	(0–2)	1	(0–2)	1	(1–2)	<.001[h]
STRATIFY score[e], median (IQR)	0	(0–1)	0	(0–0)	0	(0–1)	0	(0–1)	<.001[h]
STRATIFY score[e], mean ± SD[b]	0.42	± 0.72	0.30	± 0.56	0.39	± 0.70	0.62	± 0.89	
History of falls	807	(13.5)	201	(8.4)	255	(13.7)	351	(20.1)	<.001[i]
Mental state abnormality	353	(5.9)	76	(3.2)	94	(5.1)	183	(10.5)	<.001[i]
Vision impairment	442	(7.4)	188	(7.9)	117	(6.3)	137	(7.9)	.83[i]
Frequent urination	419	(7.0)	161	(6.8)	133	(7.2)	125	(7.2)	.59[i]
Transferring and/or mobility impairment	504	(8.4)	80	(3.4)	133	(7.2)	291	(16.7)	<.001[i]
FI-lab[f]	0.31	± 0.16	0.15	± 0.06	0.32	± 0.04	0.51	± 0.09	<.001[h]
Measured ratio	0.87	± 0.08	0.88	± 0.07	0.87	± 0.08	0.87	± 0.08	.027[h]
FI-clinical[f]	0.11	± 0.09	0.08	± 0.06	0.11	± 0.08	0.16	± 0.12	<.001[h]
Outcomes									
In-hospital falls	175	(2.9)	42	(1.8)	38	(2.0)	95	(5.5)	<.001[i]
In-hospital injurious falls	32	(0.5)	10	(0.4)	7	(0.4)	15	(0.9)	.07[i]
In-hospital mortality	95	(1.6)	3	(0.1)	8	(0.4)	84	(4.8)	<.001[i]
Length of hospital stay	9	(5–16)	8	(4–12)	8	(4–15)	12	(7–23)	<.001[h]
Discharge home	5382	(89.9)	2271	(95.5)	1718	(92.5)	1393	(80.3)	<.001[i]

Notes: Data are presented as the mean ± standard deviation, median (interquartile range), or number (percentage). CCI = Charlson Comorbidity Index; FI-clinical = standard non-laboratory Frailty Index; FI-lab = Frailty Index based on laboratory tests; IQR = interquartile range; SD = standard deviation; STRATIFY = St. Thomas's Risk Assessment Tool in Falling Elderly Inpatients.

[a]Barthel Index score ranges from 0 to 100, with a higher score indicating higher function.

[b]The mean and SD are shown for reference.

[c]The CCI score ranges from 0 to 37, with a higher score indicating more comorbidities.

[d]The polypharmacy score ranges from 0 to 6, with a higher score indicating more medications in use.

[e]The STRATIFY score ranges from 0 to 5, with a higher score indicating a higher fall risk.

[f]The FI-lab and FI-clinical scores range from 0 to 1, with a higher score indicating worse frailty.

[g]Test for trends among the three subgroups.

[h]Jonckheere-Terpstra test.

[i]Cochran-Armitage test.

Supplementary Table 8

Regression coefficients and standard errors for predictors in six prognostic models

	Model 1		Model 2		Model 3		Model 4		Model 5		Model 6	
Intercept and predictors	β	SE	β	SE	β	SE	β	SE	β	SE	β	SE
Intercept	-5.18	0.78	-5.48	0.78	-4.97	0.77	-4.85	0.77	-5.29	0.78	-5.22	0.78
Age, per year	0.02	0.01	0.01	0.01	0.01	0.01	0.01	0.01	0.01	0.01	0.01	0.01
Male sex	-0.16	0.16	-0.21	0.16	-0.18	0.16	-0.15	0.16	-0.20	0.16	-0.19	0.16
STRATIFY[a], per point	0.60	0.08	0.50	0.08			0.30	0.11			0.32	0.11
FI-lab[b], per 0.1 unit			0.24	0.05					0.21	0.05	0.21	0.05
STRATIFY including FI-lab[c], per point					0.60	0.06						
FI-clinical[b], per 0.1 unit							0.34	0.09	0.39	0.06	0.22	0.09

Notes: Each model consists of the items with values described. FI-clinical = standard non-laboratory Frailty Index; FI-lab = Frailty Index-laboratory; SE = standard error; STRATIFY = St. Thomas's Risk Assessment Tool in Falling Elderly Inpatients.

[a]The STRATIFY score ranges from 0 to 5, with a higher score indicating a higher falls risk.

[b]The FI-lab and FI-clinical scores range from 0 to 1, with a higher score indicating more severe frailty.

[c]Total of 6 items: 5 STRATIFY items and 1 item based on whether the FI-lab score was higher than 0.4.

Supplementary Table 9

Internal validation results of the model using bootstrap resampling (1000 times)

Model	Variable[a]	AUC	optimism-correted c	optimism-corrected calibration slope	mean absolute error	0.9 quantile of absolute error
Model 1	STRATIFY	0.674	0.669	0.9846	0.004	0.009
Model 2	STRATIFY + FI-lab	0.715	0.708	0.9721	0.005	0.011
Model 3	STRATIFY Including FI-lab[b]	0.718	0.714	0.9886	0.007	0.014
Model 4	STRATIFY + FI-clinical	0.707	0.700	0.9759	0.007	0.015
Model 5	FI-lab + FI-clinical	0.726	0.720	0.9775	0.007	0.016
Model 6	STRATIFY + FI-lab + FI-clinical	0.733	0.724	0.9681	0.008	0.016

Notes: AUC = area under the receiver-operating characteristic curve; FI-clinical = standard non-laboratory Frailty Index; FI-lab = Frailty Index-laboratory; STRATIFY = St. Thomas's Risk Assessment Tool in Falling Elderly Inpatients.

[a]All models include age and sex as covariates.

[b]A total of 6 items: 5 STRATIFY items and 1 item on whether FI-lab is higher than the cut-off.

Supplementary Table 10

Results of subgroup analyses. .

Subgroup	n	In-hospital fall, n (%)	ROC-AUC		
			STRATIFY	STRATIFY including FI-lab[s]	p[b]
Overall	5984	175 (2.9)	0.674	0.718	<0.001
Age					
< 75	3461	82 (2.4)	0.672	0.721	0.014
≥ 75	2523	93 (3.7)	0.647	0.698	0.002
Sex					
Male	3798	103 (2.7)	0.657	0.705	0.006
Female	2186	72 (3.3)	0.704	0.741	0.025

Notes: FI-lab = Frailty Index-laboratory; ROC-AUC = area under the receiver-operating characteristic curve; STRATIFY = St. Thomas's Risk Assessment Tool in Falling Elderly Inpatients.

[a]A total of 6 items: 5 STRATIFY items and 1 item on whether FI-lab is higher than 0.4.

[b]Comparison between STRATIFY and STRATIFY including FI-lab.

Supplementary Table 11

Results of sensitivity analyses.

Model[a]	ROC-AUC	95% CI	p
With or without polypharmacy score[b]			
STRATIFY	0.674	(0.632–0.716)	ref
STRATIFY + polypharmacy score[b]	0.675	(0.633–0.716)	0.64
STRATIFY + FI-lab	0.715	(0.677–0.753)	0.018
STRATIFY + FI-lab + polypharmacy score[b]	0.715	(0.677–0.753)	0.017
STRATIFY + FI-clinical	0.707	(0.667–0.747)	0.003
STRATIFY + FI-clinical + polypharmacy score[b]	0.709	(0.668–0.749)	0.001
With or without CCI[c]			
STRATIFY	0.674	(0.632–0.716)	ref
STRATIFY + CCI[c]	0.672	(0.630–0.715)	0.74
STRATIFY + FI-lab	0.715	(0.677–0.753)	0.018
STRATIFY + FI-lab + CCI[b]	0.715	(0.676–0.753)	0.021
STRATIFY + FI-clinical	0.707	(0.667–0.747)	0.003
STRATIFY + FI-clinical + CCI[b]	0.706	(0.666–0.747)	0.005
Changing cut-off point of FI-lab			
FI-lab (binary variable: cut-off 0.4)	0.659	(0.616–0.702)	ref
FI-lab (binary variable: cut-off 0.345)	0.670	(0.628–0.712)	0.34
STRATIFY including FI-lab[d] (binary variable: cut-off 0.4)	0.718	(0.681–0.755)	ref
STRATIFY including FI-lab[d] (binary variable: cut-off 0.345)	0.725	(0.690–0.759)	0.40
Changing fall prediction tool			
Fall risk assessment tool in the author's hospital	0.667	(0.626–0.709)	ref
STRATIFY	0.674	(0.632–0.716)	0.69
Fall risk assessment tool including FI-lab[e]	0.691	(0.652–0.731)	<0.001

Notes: CI = confidence interval; CCI = Charlson Comorbidity Index; FI-clinical = standard non-laboratory Frailty Index; FI-lab = Frailty Index-laboratory; ROC-AUC = area under the receiver-operating characteristic curve; STRATIFY = St. Thomas's Risk Assessment Tool in Falling Elderly Inpatients.

[a]All models include age and sex as covariates.

[b]The polypharmacy score ranges from 0 to 6, with a higher score indicating more medications in use.

[c]The CCI score ranges from 0 to 37, with a higher score indicating more comorbidities.

[d]A total of 6 items: 5 STRATIFY items and 1 item on whether FI-lab is higher than the cut-off.

[e]A total of 8 items: 7 items of fall risk assessment tool and 1 item on whether the FI-lab is higher than 0.4.

Supplementary Figure 1

Frequency distributions for the STRATIFY (A), standard non-laboratory Frailty Index (FI-clinical) (B), and Frailty Index-laboratory (FI-lab) (C) (n=5984). STRATIFY = St. Thomas's Risk Assessment Tool in Falling Elderly Inpatients

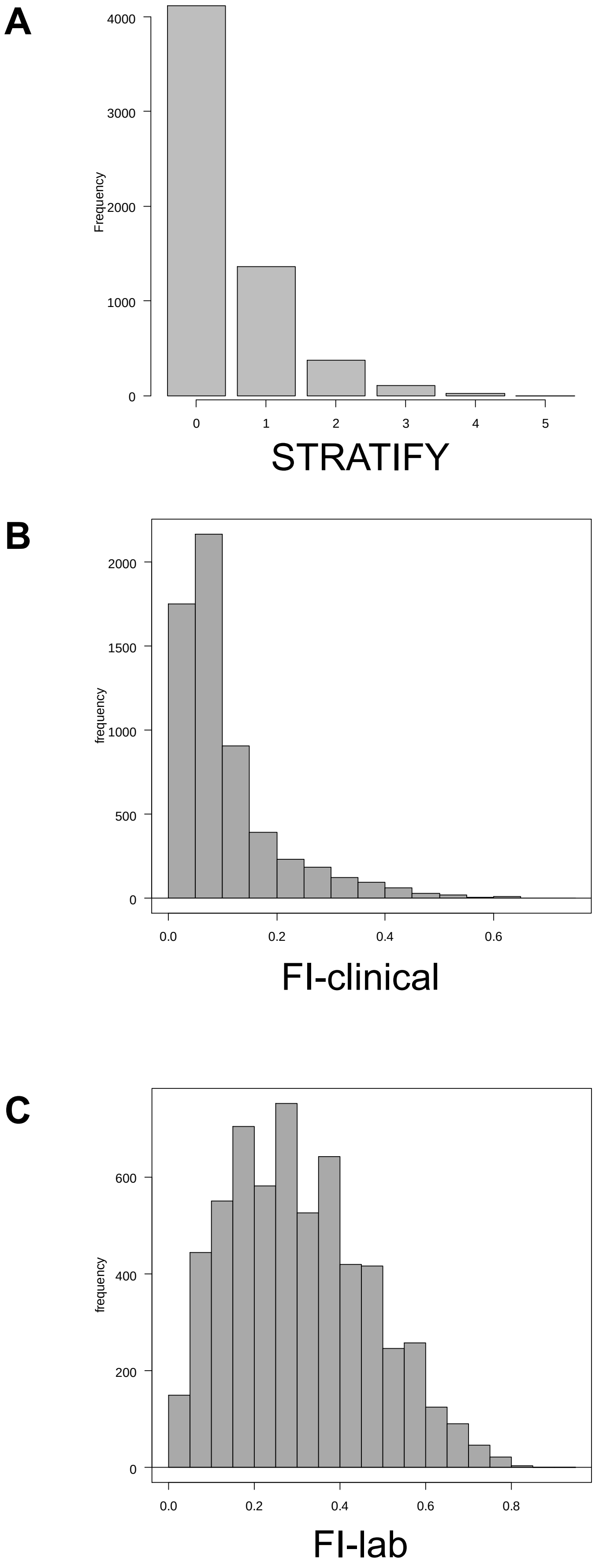

TRIPOD Checklist: Prediction Model Development

Section/Topic	Item	Checklist Item	Page
**Title and abstract**			
Title	1	Identify the study as developing and/or validating a multivariable prediction model, the target population, and the outcome to be predicted.	1
Abstract	2	Provide a summary of objectives, study design, setting, participants, sample size, predictors, outcome, statistical analysis, results, and conclusions.	2,3
**Introduction**			
Background and objectives	3a	Explain the medical context (including whether diagnostic or prognostic) and rationale for developing or validating the multivariable prediction model, including references to existing models.	4,5
	3b	Specify the objectives, including whether the study describes the development or validation of the model or both.	5
**Methods**			
Source of data	4a	Describe the study design or source of data (e.g., randomized trial, cohort, or registry data), separately for the development and validation data sets, if applicable.	5,6
	4b	Specify the key study dates, including start of accrual; end of accrual; and, if applicable, end of follow-up.	6
Participants	5a	Specify key elements of the study setting (e.g., primary care, secondary care, general population) including number and location of centres.	5
	5b	Describe eligibility criteria for participants.	6
	5c	Give details of treatments received, if relevant.	n/a
Outcome	6a	Clearly define the outcome that is predicted by the prediction model, including how and when assessed.	8
	6b	Report any actions to blind assessment of the outcome to be predicted.	8,9
Predictors	7a	Clearly define all predictors used in developing or validating the multivariable prediction model, including how and when they were measured.	7,8
	7b	Report any actions to blind assessment of predictors for the outcome and other predictors.	8,9
Sample size	8	Explain how the study size was arrived at.	10
Missing data	9	Describe how missing data were handled (e.g., complete-case analysis, single imputation, multiple imputation) with details of any imputation method.	10
Statistical analysis methods	10a	Describe how predictors were handled in the analyses.	9,10
	10b	Specify type of model, all model-building procedures (including any predictor selection), and method for internal validation.	9,10
	10d	Specify all measures used to assess model performance and, if relevant, to compare multiple models.	9,10
Risk groups	11	Provide details on how risk groups were created, if done.	9
**Results**			
Participants	13a	Describe the flow of participants through the study, including the number of participants with and without the outcome and, if applicable, a summary of the follow-up time. A diagram may be helpful.	11
	13b	Describe the characteristics of the participants (basic demographics, clinical features, available predictors), including the number of participants with missing data for predictors and outcome.	11,12
Model development	14a	Specify the number of participants and outcome events in each analysis.	11-13
	14b	If done, report the unadjusted association between each candidate predictor and outcome.	15
Model specification	15a	Present the full prediction model to allow predictions for individuals (i.e., all regression coefficients, and model intercept or baseline survival at a given time point).	Table S8
	15b	Explain how to the use the prediction model.	n/a
Model performance	16	Report performance measures (with CIs) for the prediction model.	16, 17
**Discussion**			
Limitations	18	Discuss any limitations of the study (such as nonrepresentative sample, few events per predictor, missing data).	22
Interpretation	19b	Give an overall interpretation of the results, considering objectives, limitations, and results from similar studies, and other relevant evidence.	19-22
Implications	20	Discuss the potential clinical use of the model and implications for future research.	21, 22
**Other information**			
Supplementary information	21	Provide information about the availability of supplementary resources, such as study protocol, Web calculator, and data sets.	25
Funding	22	Give the source of funding and the role of the funders for the present study.	24

We recommend using the TRIPOD Checklist in conjunction with the TRIPOD Explanation and Elaboration document.
